# Sleep and the Endocrine Brain

**DOI:** 10.1155/2010/967435

**Published:** 2011-04-04

**Authors:** Jessica A. Mong, Deborah Suchecki, Kazue Semba, Barbara L. Parry

**Affiliations:** ^1^Department of Pharmacology and Experimental Therapeutic and the Program in Neuroscience, University of Maryland School of Medicine, Baltimore, MD 21201, USA; ^2^Department of Psychobiology, Universidade Federal de São Paulo, 04024-002 São Paulo, SP, Brazil; ^3^Department of Anatomy & Neurobiology, Dalhousie University, Halifax, NS, Canada B3H 4R2; ^4^Department of Psychiatry, University of California, San Diego, CA 92037-0603, USA

Why most living organisms participate in sleep remains an enigmatic question. But in recent years, clinical and scientific studies have raised the awareness of the importance of proper sleep to overall health and quality of life. Quality sleep is imperative for the maintenance of good health. People suffering from sleep disturbances are not only fatigued but have impaired memory and learning, increased stress and anxiety, and decreased quality of daily life. While it is clear that sleep homeostasis is influenced by various neuroendocrine systems and pathological conditions, such as feeding, hormonal changes, shifts in light/dark cycles, stress, and infections to name a few, it is not clear how such conditions affect sleep homeostasis. Moreover, this is not a one-way street as neuroendocrine functions are affected by disruptions in sleep; people suffering from sleep disturbances are not only fatigued but also have impaired or dysfunctional neuroendocrine systems that affect the quality of daily life. Thus, the relationship between sleep and neuroendocrinology is an area of intense clinical and scientific interest. Understanding how neuroendocrine mediators affect sleep is central to advancing our understanding of sleep-related disorders.

The main focus of this special issue is on current findings and ideas that advance our understanding of the mechanisms underlying the neuroendocrine control of sleep and arousal. The manuscripts submitted to this special issue on sleep and the endocrine brain in the International Journal of Endocrinology center around (1) ovarian hormone control of sleep and women's health, (2) sleep and metabolism, and (3) sleep and stress. 

While much is known about the mechanics of sleep, the investigation into sex differences and hormonal control of sleep and biological rhythms is in its infancy. Data from a number of species including humans suggest that sex hormones (estrogens, progestins, and androgens) influence the physiology and pathology of sleep and biological rhythms. Women have remained underrepresented in the studies of sleep disorders even though sleep complaints are twice as prevalent in women. In recent years, more sleep studies have included women resulting in exciting findings that are raising more interesting questions. For example, while sleep complaints are generally more frequent in women, objective measures (e.g., polysomnography) suggest that women have better sleep than men. The report by A. Shechter and D.B Boivin reviews the variation in sleep and circadian rhythms at different menstrual phases in healthy women and women with premenstrual dysphoric disorder. The review by M.M. Mahoney discusses the potential consequences of disrupted biological rhythms to female reproductive functions and endocrine profiles. From these reports, it becomes clear that a better understanding of how gonadal hormones influence sleep and rhythms is necessary if we are to gain better knowledge of how dysregulation of endocrine systems influences the mechanisms of sleep and rhythm disorders.

The link between sleep loss and metabolic dysfunctions, which potentially underlies the risk for obesity and diabetes mellitus, is growing increasingly stronger. The majority of our submissions call attention to this link between sleep and metabolism. First, S. Sharma and M. Kavuru provide an in-depth overview of the research showing that sleep deprivation and sleep disorders, such as obstructive sleep apnea (OSA), have profound metabolic and cardiovascular implications. Two additional reviews focus on the associations of obstructive sleep apnea with (1) obesity, and neuroendocrine alterations in growth hormone, insulin-like growth factor-I, and the sleep-entrained prolactin rhythm (F. Lanfranco and colleagues) and (2) insulin resistance (S. Bopparaju and S. Surani). Several primary research articles investigating sleep and metabolism also are presented. Sleep duration has been inversely associated with body mass index, and M. -P. St-Onge and colleagues report gender differences in this association with their analysis of data taken from the CARDIA study. Two studies investigating sleep deprivation and glucose metabolism, one in humans and the other in rodents, present similar conclusions that sleep deprivation adversely affects glucose metabolism resulting in an increased risk for the onset of diabetes.

The hypothalamo-pituitary-adrenal (HPA) axis that controls the release of the stress hormones (cortisol in primates and corticosterone in rodents) is reciprocally connected to sleep. Sleep damps the HPA activity; however, activation of the HPA axis by a stressor is known to disrupt normal sleep patterns. In this issue, M. Balbo and colleagues discuss the potential consequences of HPA hyperactivity on sleep disturbances and the associated metabolic risks. Similarly, R.B. Machado and colleagues present findings from a rodent model of sleep deprivation that HPA-axis activation negatively impacts on sleep homeostasis. Tumors associated with the hypothalamus-pituitary axis affect endocrine functions. A clinical study by H.L. Müller in this issue reviews the association of increased daytime sleepiness and childhood craniopharyngioma. 

Our understanding of the neuroendocrine factors influencing sleep and biological rhythms is advancing. Nevertheless, more work is needed to further our understanding about the cellular and molecular mechanisms through which these factors are working. With these advances, therapeutic targets may be elucidated that will help to alleviate the sleep pathologies associated with neuroendocrine dysfunctions. 

*Jessica A. Mong* *Jessica A. Mong* *Deborah Suchecki* *Deborah Suchecki* *Kazue Semba* *Kazue Semba* *Barbara L. Parry* *Barbara L. Parry*

